# Potential roles of the gut microbiota in the manifestations of drug use disorders

**DOI:** 10.3389/fpsyt.2022.1046804

**Published:** 2022-12-13

**Authors:** Zhiyan Wang, Chengqian Hou, Lei Chen, Mingming Zhang, Wenbo Luo

**Affiliations:** ^1^Research Center of Brain and Cognitive Neuroscience, Liaoning Normal University, Dalian, China; ^2^Key Laboratory of Brain and Cognitive Neuroscience, Dalian, China

**Keywords:** drug use disorders, gut microbiota, gut-brain axis, methamphetamine, cocaine, opioids

## Abstract

Drug use disorders (DUDs) not only cause serious harm to users but also cause huge economic, security, and public health burdens to families and society. Recently, several studies have shown that gut microbiota (GM) can affect the central nervous system and brain functions. In this review, we focus on the potential role of the GM in the different stages of DUDs. First, the GM may induce individuals to seek novel substances. Second, the gut microbiota is involved in the decomposition and absorption of drugs. Symptoms of individuals who suffer from DUDs are also related to intestinal microorganisms. Third, the effects of the GM and its metabolites on drug relapse are mainly reflected in the reward effect and drug memory. In conclusion, recent studies have preliminarily explored the relationship between GM and DUDs. This review deepens our understanding of the mechanisms of DUDs and provides important information for the future development of clinical treatment for DUDs.

## Introduction

Drug use disorders (DUDs) are notable as a global public health problem. According to the latest report from the United Nations Office on Drugs and Crime, approximate 284 million people aged 15–64 years old worldwide have used drugs in 2020 (1). Notably, people with DUDs who are co-infected with the severe acute respiratory syndrome coronavirus 2, the etiological agent of the current coronavirus disease 2019 pandemic, are at risk of contracting multiple diseases. In addition, because of the hindered economic development in various countries due to the pandemic, the possibility of drug use by high-risk groups and the risk of relapse by addicts during the abstinence period will significantly increase (2, 3). DUDs significantly harm the physical and mental health of drug abusers, and it is associated with public health and safety problems (e.g., AIDS) (4). Importantly, DUDs are considered the chronic and recurrent brain disease, which can impair brain function by rebuilding reward pathways and changing synaptic plasticity (5). At present, the treatment of DUDs mainly depends on psychological withdrawal and drug substitution, but new treatment ideas are urgently required (6).

Recently, researchers found that the gut microbiota (GM) is involved in the pathogenesis and progression of many diseases. The intestinal microflora is a complex microbial ecosystem, which is balanced by the interaction of internal and external environments and maintains the normal physiological functions of the host (7, 8). The GM may communicate with the brain through the intestinal and autonomic nervous systems and the immune system, thereby affecting brain function (9). The signal transduction pathway between biochemical molecules and neurons is known as the microbial-intestinal-brain axis (10). In addition, the GM participates in the synthesis of neuroactive molecules (e.g., dopamine and 5-hydroxytryptamine) and affects the central nervous system (CNS) by activating the vagus nerve, stimulating the immune pathway, and inducing signal transduction in the intestinal nervous system (11, 12). Therefore, the GM may be an important factor in many neurological diseases (10, 13). Li et al. (14) conducted a random-effects meta-analysis on the standardized mean difference of intestinal microbial diversity by using community richness, community diversity, and phylogenetic diversity. It was found that the alpha diversity of intestinal microbiota may be an effective predictor of neurological diseases such as Alzheimer’s disease, schizophrenia, and multiple sclerosis. In another meta-analysis study of general adult people with mental illness, the intestinal microbial richness of patients decreased significantly in terms of alpha diversity compared with the control group. The differences in beta diversity could be observed in major depressive disorder and psychosis and schizophrenia (15). These suggest that psychiatric disorders may be associated with a unique pattern of microbial perturbations, which may be used as a biomarker.

As a neurological brain disease, do DUDs also relate to GM? If so, what are the potential roles of GM in the different stages of DUDs? The effects of the gut microbes on DUDs are subtle yet extensive, which may have been overlooked in previous studies. The present review systematically summarizes recent findings and discusses the role of the GM in different DUDs stages. The current review will have important implications for exploring the mechanisms of DUDs development. In addition, this review will highlight potential diagnoses and therapeutic options.

## Gut microbiota and novelty seeking

The initiation of drug use is closely related to family environment and social factors (e.g., parental influences and deviant friends) (16). There is also a positive correlation between the frequency of addictive substance use and the level of sensation seeking, which could be one predictor of drug use (17). However, the development of DUDs mainly depends on neuropharmacology and neurobiological factors (18). The GM plays a role in the initial stage of DUDs, especially in the seeking and preference of novel substances.

Specifically, people who pursue high novelty tend to be more sensitive to novel, packaged addictive drugs. Individuals with cocaine and methamphetamine (MA) use disorder have significantly higher levels of drug seeking than healthy controls, which may impact drug use (19). There is also a positive correlation between novelty seeking and relapse of cocaine users (20). Novelty seeking can partially predict individual drug susceptibility and abuse trends, and this has clinical value in the prevention of DUDs (21). Interestingly, some changes in the GM can significantly enhance the novelty-seeking behavior of animals. For example, intervention with a high-dose probiotic mixture can increase the number of *Bifidobacteria* in ferrets, thus increasing the time spent interacting with the novelty kettlebell and “strange animals” (22). In addition, other studies have shown that antibiotic-treated mice have decreased gut microbial content, resulting in a significant increase in their preference for cocaine, suggesting that the GM and its metabolites might enhance individual preference for cocaine (23–25).

The GM could also indirectly increase the possibility of using addictive drugs through ghrelin, a hormone produced by the gastrointestinal tract. Positive associations have been observed between ghrelin and total bacteria, *Clostridium*, and *Ruminococcus*; a negative association between an increased *Bacteroidets/Firmicutes* ratio, *Faecalibacterium*, *Prevotellaceae*, and ghrelin levels has also been found (26). For example, the abundance of *Proteus*, *Bacteroides*, *Clostridium*, and *Prevotella* in rats fed a restricted diet was significantly higher than in rats fed an unrestricted diet, while the number of actinomycetes, thick-walled phyla, *Lactobacillus*, and *Bifidobacterium* decreased significantly (27). The single-generation metabolites or related derivatives (e.g., short-chain fatty acids; SCFAs) of GM may be the key inducer or driving force of gut-brain communication (28). Rahat-Rozenbloom et al. (29) also found that ghrelin secretion decreased with an acute increase in SCFAs. Notably, upregulation of the ghrelin system may also increase individual cravings for drug use (30). It is closely linked to the central dopamine system and can promote the expression of dopamine receptors in the striatum, enhance reward behavior, and enhance novelty-seeking behavior in rodents (31, 32). Hansson et al. (33) also observed that the injection of ghrelin into rats increased their exploration of novel targets, and these rats showed a stronger preference for the new environment. Conversely, when the ghrelin receptor was inhibited, the novel response of rats was significantly weakened. These authors also collected venous blood from human subjects and grouped genes encoding ghrelin and gastrin receptors. The results showed that ghrelin receptor antagonists weakened individuals’ preference for a new environment and decreased novelty activity. There was a significant negative correlation between ghrelin receptor single nucleotide polymorphism and novelty traits of the subjects. Therefore, GM and its metabolites could interfere with the level of ghrelin secretion, which may have a further effect on some external behaviors (e.g., novelty seeking) of individuals to some extent. That is, the effect of the GM on ghrelin may also be an important basis for novelty seeking in rodents and humans.

Taken together, GM and its metabolites could change individual’s preference for novel substances in difference degrees through both direct and indirect ways, especially for the people with high susceptibility to addictive drugs. In the future, GM interventions for susceptible drug users may reduce the possibility of first use of addictive drugs.

## Gut microbiota and drug taking

### Drug metabolism by gut microbiota

A large number and variety of intestinal flora in the human body participate in the physiological activities of the host in many forms. With the progress of biotechnology, the intestinal microflora has been gradually regarded as another “metabolic organ” of the body, and its metabolic ability is comparable to that of the liver. Previous studies have shown that the intestinal flora could catabolize drugs (34, 35). Specifically, the GM directly affects *in vivo* drug metabolism and induces biotransformation reactions (e.g., demethylation and dehydroxylation) (36). In some conjugate hydrolysis reactions to drug toxicity, the GM may indirectly affect drug toxicity levels by regulating the competition between bacterial metabolites in different metabolic pathways (36).

Moreover, the GM may affect the decomposition and absorption of MA. Salamanca et al. (37) reviewed that although MA metabolism mainly depends on the liver, its primary and secondary metabolites are absorbed by the gastrointestinal tract for further metabolic activities. Second, individuals who used MA showed symptoms of acute transient ischemic colitis the following day, indicating that MA can be decomposed and absorbed by the human GM and various digestive enzymes, causing damage to the intestinal environment, and resulting in symptoms of intestinal disease (38). In addition, Caldwell et al. (39) showed that *Lactobacillus*, *Enterococcus*, and *Clostridium* in guinea pig intestines can transform MA through *N*-demethylation and other ways.

Similarly, the GM is also involved in the catabolism of opioids. The plasma concentration of morphine is lower in cancer patients after oral morphine administration than in cancer patients after rectal morphine administration; this may be due to morphine catabolism by the GM (40). Wang et al. (41) also revealed that microbes (e.g., *Bacteroides* and *Bifidobacterium*) could regulate the reabsorption of morphine by expressing β-glucuronidase; the loss of catabolic metabolites (e.g., those of *Bacteroides*) regulated morphine metabolism and enterohepatic recycling. Therefore, the intervention of GM could decrease the absorption rate of morphine to weaken effect of analgesia. This is also the main reason why long-term use of morphine will lead to tolerance and drug addiction.

Taken together, these results suggest that the GM may have a high metabolic ability for addictive drugs. As many factors can affect microbial metabolism (e.g., drug type, host, and microbiome differences), researchers are also considering combining chemistry and toxicology to predict the effects of the GM on drug metabolism and toxicity. For example, Guthrie et al. (42) proposed a graph database called the MicrobeFDT, which clusters chemically similar drug and food compounds and links these compounds to microbial enzymes and known toxicities. This set could be used to study and predict the contribution of microbial *N*-demethylase to drug metabolism and toxicity. In addition, future research may reduce drug-induced CNS damage by using the gut-brain axis through GM intervention. An in-depth understanding of the effect of the GM on the metabolism of addictive drugs is of great significance in guiding toxicological research and the clinical treatment of DUDs.

### Influence of addictive drugs on gut microbiota

Many animal and human experiments have also indicated that addictive drugs can alter the diversity of the GM. Scorza et al. (43) showed that the abundance of *Spirochetaceae* and *Desulfovibrionaceae* in cocaine-treated rat feces diminished significantly, while the abundance of *Lachnospiraceae* and *Prevotellaceae* increased. There is a significant ecological imbalance in the fecal microbial population of chronic opioid users. Another study found that the relative abundance of *Bacteroidaceae* in the gut of patients with cirrhosis receiving opioid treatment was significantly lower than that in patients with cirrhosis not receiving opioids (44). Additionally, Barengolts et al. (45) detected *Bifidobacterium* in fecal samples from male patients with type 2 diabetes and found that their abundance in opioid users was significantly higher than that in non-users. This may be because opioids affect the abundance of bifidobacteria when they are used as organic cation transporter 1 inhibitors. Studies on drug users have also shown that there are differences in GM diversity between opioid users and healthy individuals. For example, the relative abundance of *Roseburia* and *Bilophila* was lower in participants who used opioid agonists compared to participants who used neither opioid agonists nor antagonists (46). Therefore, cocaine and opioids may cause dysbiosis in the intestinal environment.

These gut microbial changes further affect brain function and have long-term effects on the CNS of the user. Of note, various symptoms associated with DUDs (e.g., emotional disorders, increased susceptibility to drugs, and brain damage) are directly or indirectly related to GM. First, changes in the GM are closely related to the various mental symptoms associated with DUDs. Forouzan et al. (47) found that after injection of MA, the fecal samples of rats in the experimental (MA) group had a higher diversity of *Actinomycetes* and a lower diversity of *Bacteroides* than in the fecal samples of rats in the control (saline) group. The relative abundances of *Bifidobacterium* and *Lactobacillus* in the experimental group were higher than in the control group, and the exploratory behavior of rats in the MA group decreased during the withdrawal period, accompanied by depressive behavior. Ning et al. (48) investigated the GM of rats with conditioned location preference in the MA and control groups using 16SrRNA and high-throughput sequencing. Their results showed that the abundance of *Coccidiaceae* in the control group was higher than that in the experimental group, while that of *Verruciaceae* and *Bacillus* in the experimental group was higher than that in the control group. Verrucous microflora in the intestinal microflora is associated with anxiety (49). Recently, Yang et al. (50) detected the fecal samples of MA users and found that the overall Shannon diversity index of GM in the addiction group was lower than that in the healthy group; the *Enterobacteriaceae* diversity in the addiction group was positively correlated with delusions, suspicions, and other mental symptoms, and the total general psychopathology scale was negatively correlated with the abundance of *Collinsella* and *Faecalibacterium*. Therefore, the long-term mental symptoms of DUDs are closely related to GM and its metabolites.

Second, GM changes are related to the preference and sensitivity for addictive drugs. For example, Yang et al. (51) revealed that after the MA-induced conditioned place preference (CPP) paradigm, the CPP score was positively correlated with the relative abundance of *Verrucomicrobia* (phylum) and *Verrucomicrobiaceae* (family). They also showed that the CPP score of antibiotic-treated rats was significantly higher than that of rats treated with distilled water, and the former had an increased preference for MA. Repeated use of morphine could cause the decrease of *Olsenella* and *Rothia*, and the increase of *Helicobacter*, which may have a higher risk of addictive behaviors (52). The differential relative abundance of these taxa may be the nature of rats with high/low sensitivity to morphine. The disorder of intestinal microbiota may improve the sensitivity of users to drugs and increase their preference for drugs. Therefore, the GM may be an important intrinsic factor in addictive drug-induced behavioral changes and DUDs.

Finally, the GM changes induced by DUDs are also associated with neurotoxicity and brain injury in DUD patients. The fecal samples of mice treated with multiple high doses of MA had a higher diversity of GM species than those of the control group, particularly, a reduced relative abundance of *Lactobacillaceae* and *Prevodiaceae* and an increased relative abundance of *Pseudomonas* and *Enterobacteriaceae*; compared with the control group, the expression of monoamine oxidase in the striatum of MA-treated mice increased significantly, while the expression of tyrosine hydroxylase decreased, which indicates that MA could induce dopamine terminal neurotoxicity (53). Drug use can change the diversity of intestinal flora and increase the permeability of the blood-brain barrier. In certain cases, these have pathogenic intestinal microflora and its secretions can enter the brain and induce neurotoxicity. Choi et al. (54) also showed that an increase in the abundance of *Enterobacteriaceae* may be involved in the damage of dopaminergic neurons and inflammation in the substantia nigra and striatum. Cook et al. (55) sequenced the *16SrRNA* gene from human male rectal swab samples and found that MA abuse was associated with significant changes in overall composition of the gastrointestinal microbiome (e.g., *Parvimonas*, *Butyricicoccus*, and *Faecalibacterium*), which also included some pathogenic bacteria with neural activity potential. Therefore, after addictive drug intake, pathogenic GM and its metabolites may enter the CNS through damaged barriers and signaling pathways, resulting in brain dysfunction.

To some extent, many of the above studies indicate that the decomposition and absorption of addictive drugs can affect the intestinal microbiota of the body. The frequent use of addictive drugs intensifies their influence on GM, which will have lasting effects on users’ psychological and physiological functions. In addition, the gut sends signals to the brain through the production of neuroactive metabolites, signaling *via* the vagus nerve, and interactions with the immune system (56, 57). This is a new way to understand the relationship between the GM and DUDs. Further elucidation of the interaction between the GM and the immune system may contribute to a deeper exploration of the mechanisms of DUDs.

## Gut microbiota and drug relapse

Compulsive drug use is one of the core features of DUDs, causing some individuals to relapse after withdrawal. The neurobiochemical mechanism of relapse after withdrawal is mainly reflected in changes in synapses in the brain caused by using drugs (58–60). These changes mainly include the reward mechanism and drug memory, which are closely related to relapse. The abnormal connection between drugs and the reward mechanism is manifested in the activity of neurons in multiple brain regions. In addition, after abstinence, drug-related episodic memory and cues can activate dopamine neurons and promote an individual’s sense of craving and seeking behavior. In the relapse process of drug addiction, GM and its derivatives also play an invisible role in the memory and reward mechanism of drug addiction.

### Gut microbiota and its metabolites in drug reward

The GM and its metabolites are directly involved in the reward mechanisms of addictive drugs. The rewarding effect is activated by dopamine neurons located in the ventral tegmental area, which project to areas such as the nucleus accumbens. The release of dopamine and other neurotransmitters promotes euphoria (61). The GM may act on the brain area of dopaminergic nerve transmission, and the dopamine circuit is sensitive to these changes (62). There is increasing evidence that the microbial-gut-brain axis may be a key factor in regulating the reward mechanism and is closely related to the occurrence of related diseases (63). Lee et al. (25) found that there was a causal relationship between the changes in GM and neuroinflammation and impaired reward response in mice treated with antibiotics. Moreover, the normal reward behavior of mice could be restored by fecal microbial transplantation. The GM and its metabolites are also necessary for the morphine reward mechanism. Hofford et al. (24) showed that the diversity of the GM and the SCFA levels decreased in antibiotic-treated mice, showing a persistent weakening of the reward effect of morphine because a decrease in diversity changes the transcriptional response of morphine in the nucleus accumbens. However, supplementation with SCFAs can reverse morphine reward defects caused by antibiotics. Therefore, the GM and its metabolites play important roles in the reward mechanism of addictive drugs.

In addition, some previous work has also suggested that the GM and its metabolites could indirectly regulate drug-related reward pathway *via* glucagon-like peptide 1 (GLP-1). GLP-1, produced in the gastrointestinal tract, is encoded by the glucagon gene, and its receptor (GLP-1R) is widely found in the CNS. The change of GLP-1 levels was correlated with the disturbances experienced by different families, genera, and species of the microbiota. For example, some families, genera, and species of the phylum *Actinobacteria* and *Firmicutes* showed positive correlations with GLP-1 levels, while the families and genera of the phylum *Bacteroidetes* and the species *Blautia producta* have the opposite correlations with GLP-1 levels (64). Neurons expressing GLP-1 can project to areas of the brain associated with reward (e.g., the ventral tegmental area and nucleus accumbens) (65). The metabolite SCFAs of GM may stimulate the release of GLP-1 through the phospholipase C signaling pathway (66). Breton et al. (67) found that infusion of *Escherichia coli* proteins into the rat colon could also stimulate the secretion of GLP-1 and increase its concentration in the plasma. Moreover, GLP-1 and its analogs could regulate abnormal reward effects caused by drugs (e.g., cocaine, amphetamine); its receptors are expressed in the reward-related areas (68). GLP-1 and GLP-1R enhance the behavioral response of mice to cocaine, and the loss of GLP-1R could regulate the anxiety-related behavior (69). Another study showed that activation of GLP-1R in the ventral tegmental area attenuates cocaine intake in rats (70). GLP-1R antagonists reduced the self-administration behavior and recurrence behavior of MA withdrawn rats (71). The above evidence shows that GLP-1 and GLP-1R play important roles in drug relapse.

During relapse, the GM directly or indirectly enhances the reward effect of drug use. Therefore, the GM contributes to the neural mechanism of the individual pursuit of pleasure. Drugs change the GM composition and act on the CNS, and this may be the potential connection between the GM and relapse after withdrawal; microbiological therapy for this abnormal connection may reduce DUDs relapses.

### Gut microbiota and drug memory

The drug-related stimuli can trigger memories of addiction and lead to re-use of the drug, so drug memory is an important factor for relapse (72, 73). Although there is not a lot of evidence on the direct relationship between GM and drug addiction memory, the existing research is still suggestive.

The following studies suggest that gut microbes may influence memory function through lactic acid produced by astrocytes. Astrocytes can regulate the neuronal activity, synaptic transmission, and plasticity by providing energy and growth factors and producing neurotransmitters (74). The lactic acid is transported to the interstitial fluid *via* monocarboxylic acid transporter (MCT)1/4 and transported into the neuron *via* MCT2 (74, 75). GM and its metabolites (e.g., SCFAs, glutamate) affect the production and transport of lactic acid in astrocytes (28, 76). Lactic acid release in astrocytes is necessary for the development and maintenance of long-term memory. Lactic acid is involved in synaptic plasticity, memory formation, and signal transduction in DUDs (77). For example, lactic acid in astrocytes regulates synaptic plasticity, and its release contributes to the formation of cocaine memory (78). Zhang et al. (79) also found that as the concentration of lactic acid in the basolateral amygdala decreased, cocaine use also decreased in rats. This suggests that the transport of lactic acid regulated by GM and its derivations is important for the drug memory.

Taken together, as a signaling molecule, lactic acid in astrocytes is important to the drug memory (80). Although little research provides the evidence that GM can directly affect drug memory, a previous study has shown that memory disorders in rats with elevated levels of lactic acid, an important biomarker, could be improved by correcting intestinal microbial disorders (e.g., *Lactobacilllus*, *Bacteroidales*, and *Bacteroides*) (81, 82). Therefore, future research on drug memory and reducing relapse may be carried out through GM therapy to interfere with the production and secretion of lactic acid.

## Conclusion

It can be seen from this review that GM and its metabolites play a considerable role in the different stages of drug addiction. First, when the GM of individuals with high susceptibility to addictive drugs changes, they tend to seek novel substances. Second, the GM could interfere with the breakdown and adsorption of drugs in the body at the beginning of using. Third, during the withdrawal period, the intervention of intestinal microbes may interfere with the formation of drug memory to a certain extent, which is of great significance for the prevention of relapse among addictive patients.

The limitations of current technology challenge research progress but also bring opportunities. Importantly, it is helpful to explore the process of microecological circulation in the human body and reveal the mechanisms employed by the GM in individual physiology and psychology. Some researchers have pointed out that microbiological medicine is a new medical model in the 21st century (83). The microbiota will become the frontier and center of disease prevention and treatment (84). Microbiological therapy may be a more comprehensive and multi-effect approach for some chronic and recurrent diseases (85, 86). This is beneficial to the development of drugs for the treatment of DUDs. Therefore, future research may reduce drug-induced damage by using the gut-brain axis through GM intervention.

## Author contributions

ZW contributed to conceptualizing the work, drafting the manuscript, and managing the project. WL, MZ, CH, and LC provided support for conceptualizing and drafting the manuscript. WL and MZ supervised the project. All the authors reviewed and edited the manuscript.

## References

[1] United Nations Office on Drugs and Crime. *World Drug Report 2022.* New York, NY: United Nations (2022).

[2] Volkow N. Collision of the COVID-19 and addiction epidemics. *Ann Intern Med.* (2020) 173:61–2. 10.7326/M20-1212 32240293 PMC7138334

[3] Cisneros I Cunningham K. Covid-19 interface with drug misuse and substance use disorders. *Neuropharmacology.* (2021) 198:108766. 10.1016/j.neuropharm.2021.108766 34454912 PMC8388132

[4] Leshner A. Addiction is a brain disease, and it matters. *Science.* (1997) 278:45–7. 10.1126/science.278.5335.45 9311924

[5] Cheron J d’Exaerde A. Drug addiction: from bench to bedside. *Transl Psychiatry.* (2021) 11:424. 10.1038/s41398-021-01542-0 34385417 PMC8361217

[6] Tyndale R. Drug addiction: a critical problem calling for novel solutions. *Clin Pharmacol Ther.* (2008) 83:503–6. 10.1038/clpt.2008.11 18349865

[7] Clemente J Ursell L Parfrey L Knight R. The impact of the gut microbiota on human health: an integrative view. *Cell.* (2012) 148:1258–70. 10.1016/j.cell.2012.01.035 22424233 PMC5050011

[8] Stilling R Dinan T Cryan J. Microbial genes, brain & behaviour - epigenetic regulation of the gut-brain axis. *Genes Brain Behav.* (2014) 13:69–86. 10.1111/gbb.12109 24286462

[9] Banks W. The blood-brain barrier: connecting the gut and the brain. *Regul Pept.* (2008) 149:11–4. 10.1016/j.regpep.2007.08.027 18486244 PMC2553040

[10] Agirman G Yu K Hsiao E. Signaling inflammation across the gut-brain axis. *Science.* (2021) 374:1087–92. 10.1126/science.abi6087 34822299

[11] Nagpal J Cryan J. Microbiota-brain interactions: moving toward mechanisms in model organisms. *Neuron.* (2021) 109:3930–53. 10.1016/j.neuron.2021.09.036 34653349

[12] Simpson S Mclellan R Wellmeyer E Matalon F George O. Drugs and bugs: the gut-brain axis and substance use disorders. *J Neuroimmune Pharmacol.* (2021). 10.1007/s11481-021-10022-7 [Epub ahead of print]. 34694571 PMC9074906

[13] Cryan J O’Riordan K Sandhu K Peterson V Dinan T. The gut microbiome in neurological disorders. *Lancet Neurol.* (2020) 19:179–94. 10.1016/S1474-4422(19)30356-4 31753762

[14] Li Z Zhou J Liang H Ye L Lan L Lu F Differences in alpha diversity of gut microbiota in neurological diseases. *Front Neurosci.* (2022) 16:879318. 10.3389/fnins.2022.879318 35837118 PMC9274120

[15] Nikolova V Smith M Hall L Cleare A Stone J Young A. Perturbations in gut microbiota composition in psychiatric disorders: a review and meta-analysis. *JAMA Psychiatry.* (2021) 78:1343–54. 10.1001/jamapsychiatry.2021.2573 34524405 PMC8444066

[16] Mirnics Z Kövi Z Tanyi Z Grezsa F. Adolescent drug use, relational variables and personality factors. *Psychiatr Danub.* (2021) 33:656–65. 34718295

[17] Hamdan-Mansour A Mahmoud K Shibi A Arabiat D. Impulsivity and sensation-seeking personality traits as predictors of substance use among university students. *J Psychosoc Nurs Ment Health Serv.* (2018) 56:57–63. 10.3928/02793695-20170905-04 28892553

[18] Glantz M Pickens R. *Vulnerability to Drug Abuse.* Washington, DC: American Psychological Association (1992).

[19] Mahoney J Thompson-Lake D Cooper K Verrico C Newton T De La Garza R II. A comparison of impulsivity, depressive symptoms, lifetime stress and sensation seeking in healthy controls versus participants with cocaine or methamphetamine use disorders. *J Psychopharmacol.* (2015) 29:50–6. 10.1177/0269881114560182 25424624

[20] Ismael F Baltieri D. Role of personality traits in cocaine craving throughout an outpatient psychosocial treatment program. *Braz J Psychiatry.* (2014) 36:24–31. 10.1590/1516-4446-2013-1206 24604459

[21] Wingo T Nesil T Choi J Li M. Novelty seeking and drug addiction in humans and animals: from behavior to molecules. *J Neuroimmune Pharmacol.* (2016) 11:456–70. 10.1007/s11481-015-9636-7 26481371 PMC4837094

[22] Dugyala S Ptacek T Simon J Li Y Frohlich F. Putative modulation of the gut microbiome by probiotics enhances preference for novelty in a preliminary double-blind placebo-controlled study in ferrets. *Anim Microbiome.* (2020) 2:14. 10.1186/s42523-020-00030-y 32490353 PMC7266289

[23] Kiraly D Walker D Calipari E Labonte B Issler O Pena C Alterations of the host microbiome affect behavioral responses to cocaine. *Sci Rep.* (2016) 6:35455. 10.1038/srep35455 27752130 PMC5067576

[24] Hofford R Mervosh N Euston T Meckel K Orr A Kiraly D. Alterations in microbiome composition and metabolic byproducts drive behavioral and transcriptional responses to morphine. *Neuropsychopharmacology.* (2021) 46:2062–72. 10.1038/s41386-021-01043-0 34127799 PMC8505488

[25] Lee K Vuong H Nusbaum D Hsiao E Evans C Taylor A. The gut microbiota mediates reward and sensory responses associated with regimen-selective morphine dependence. *Neuropsychopharmacology.* (2018) 43:2606–14. 10.1038/s41386-018-0211-9 30258112 PMC6224506

[26] Leeuwendaal N Cryan J Schellekens H. Gut peptides and the microbiome: focus on ghrelin. *Curr Opin Endocrinol Diabetes Obes.* (2021) 28:243–52. 10.1097/MED.0000000000000616 33481425 PMC7924980

[27] Queipo-Ortuno M Seoane L Murri M Pardo M Gomez-Zumaquero J Cardona F Gut microbiota composition in male rat models under different nutritional status and physical activity and its association with serum leptin and ghrelin levels. *PLoS One.* (2013) 8:e65465. 10.1371/journal.pone.0065465 23724144 PMC3665787

[28] Ahmed H Leyrolle Q Koistinen V Kärkkäinen O Layé S Delzenne N Microbiota-derived metabolites as drivers of gut-brain communication. *Gut Microbes.* (2022) 14:2102878. 10.1080/19490976.2022.2102878 35903003 PMC9341364

[29] Rahat-Rozenbloom S Fernandes J Cheng J Wolever T. Acute increases in serum colonic short-chain fatty acids elicited by inulin do not increase GLP-1 or PYY responses but may reduce ghrelin in lean and overweight humans. *Eur J Clin Nutr.* (2017) 71:953–8. 10.1038/ejcn.2016.249 27966574 PMC5423780

[30] Zallar L Farokhnia M Tunstall B Vendruscolo L Leggio L. The role of the ghrelin system in drug addiction. *Int Rev Neurobiol.* (2017) 136:89–119. 10.1016/bs.irn.2017.08.002 29056157

[31] Abizaid A Liu Z Andrews Z Shanabrough M Borok E Elsworth J Ghrelin modulates the activity and synaptic input organization of midbrain dopamine neurons while promoting appetite. *J Clin Invest.* (2006) 116:3229–39. 10.1172/JCI29867 17060947 PMC1618869

[32] Jerlhag E Egecioglu E Dickson S Douhan A Svensson L Engel J. Ghrelin administration into tegmental areas stimulates locomotor activity and increases extracellular concentration of dopamine in the nucleus accumbens. *Addict Biol.* (2007) 12:6–16. 10.1111/j.1369-1600.2006.00041.x 17407492

[33] Hansson C Shirazi R Naslund J Vogel H Neuber C Holm G Ghrelin influences novelty seeking behavior in rodents and men. *PLoS One.* (2012) 7:e50409. 10.1371/journal.pone.0050409 23227170 PMC3515575

[34] Mikov M. The metabolism of drugs by the gut flora. *Eur J Drug Metab Pharmacokinet.* (1994) 19:201–7. 10.1007/BF03188922 7867662

[35] Li H He J Jia W. The influence of gut microbiota on drug metabolism and toxicity. *Expert Opin Drug Metab Toxicol.* (2016) 12:31–40. 10.1517/17425255.2016.1121234 26569070 PMC5683181

[36] Wilson I Nicholson J. Gut microbiome interactions with drug metabolism, efficacy, and toxicity. *Transl Res.* (2017) 179:204–22. 10.1016/j.trsl.2016.08.002 27591027 PMC5718288

[37] Salamanca S Sorrentino E Nosanchuk J Martinez L. Impact of methamphetamine on infection and immunity. *Front Neurosci.* (2015) 8:445. 10.3389/fnins.2014.00445 25628526 PMC4290678

[38] Herr R Caravati E. Acute transient ischemic colitis after oral methamphetamine ingestion. *Am J Emerg Med.* (1991) 9:406–9. 10.1016/0735-6757(91)90073-s 2054019

[39] Caldwell J Hawksworth G. The demethylation of methamphetamine by intestinal microflora. *J Pharm Pharmacol.* (1973) 25:422–4. 10.1111/j.2042-7158.1973.tb10043.x 4146404

[40] Du X Skopp G Aderjan R. The influence of the route of administration: a comparative study at steady state of oral sustained release morphine and morphine sulfate suppositories. *Ther Drug Monit.* (1999) 21:208–14. 10.1097/00007691-199904000-00011 10217341

[41] Wang F Meng J Zhang L Johnson T Chen C Roy S. Morphine induces changes in the gut microbiome and metabolome in a morphine dependence model. *Sci Rep.* (2018) 8:3596. 10.1038/s41598-018-21915-8 29483538 PMC5827657

[42] Guthrie L Wolfson S Kelly L. The human gut chemical landscape predicts microbe-mediated biotransformation of foods and drugs. *Elife.* (2019) 8:e42866. 10.7554/eLife.42866 31184303 PMC6559788

[43] Scorza C Piccini C Martinez Busi M Abin Carriquiry J Zunino P. Alterations in the gut microbiota of rats chronically exposed to volatilized cocaine and its active adulterants caffeine and phenacetin. *Neurotox Res.* (2019) 35:111–21. 10.1007/s12640-018-9936-9 30066173

[44] Acharya C Betrapally N Gillevet P Sterling R Akbarali H White M Chronic opioid use is associated with altered gut microbiota and predicts readmissions in patients with cirrhosis. *Aliment Pharmacol Ther.* (2017) 45:319–31. 10.1111/apt.13858 27868217

[45] Barengolts E Green S Eisenberg Y Akbar A Reddivari B Layden B Gut microbiota varies by opioid use, circulating leptin and oxytocin in African American men with diabetes and high burden of chronic disease. *PLoS One.* (2018) 13:e0194171. 10.1371/journal.pone.0194171 29596446 PMC5875756

[46] Gicquelais R Bohnert A Thomas L Foxman B. Opioid agonist and antagonist use and the gut microbiota: associations among people in addiction treatment. *Sci Rep.* (2020) 10:19471. 10.1038/s41598-020-76570-9 33173098 PMC7655955

[47] Forouzan S Hoffman K Kosten T. Methamphetamine exposure and its cessation alter gut microbiota and induce depressive-like behavioral effects on rats. *Psychopharmacology.* (2021) 238:281–92. 10.1007/s00213-020-05681-y 33097978

[48] Ning T Gong X Xie L Ma B. Gut microbiota analysis in rats with methamphetamine-induced conditioned place preference. *Front Microbiol.* (2017) 8:1620. 10.3389/fmicb.2017.01620 28890714 PMC5575146

[49] Tarr A Galley J Fisher S Chichlowski M Berg B Bailey M. The prebiotics 3’Sialyllactose and 6’Sialyllactose diminish stressor-induced anxiety-like behavior and colonic microbiota alterations: Evidence for effects on the gut–brain axis. *Brain Behav Immun.* (2015) 50:166–77. 10.1016/j.bbi.2015.06.025 26144888 PMC4631662

[50] Yang Y Yu X Liu X Liu G Zeng K Wang G. Altered fecal microbiota composition in individuals who abuse methamphetamine. *Sci Rep.* (2021) 11:18178. 10.1038/s41598-021-97548-1 34518605 PMC8437956

[51] Yang C Fu X Hao W Xiang X Liu T Yang B Gut dysbiosis associated with the rats’ responses in methamphetamine-induced conditioned place preference. *Addict Biol.* (2021) 26:e12975. 10.1111/adb.12975 33094505

[52] Zhang J Yang J Yang C Chen T Wang Z Li J Sensitivity to morphine reward associates with gut dysbiosis in rats with morphine-induced conditioned place preference. *Front Psychiatry.* (2020) 11:631. 10.3389/fpsyt.2020.00631 33005148 PMC7484999

[53] Chen L Zhi X Zhang K Wang L Li J Liu J Escalating dose-multiple binge methamphetamine treatment elicits neurotoxicity, altering gut microbiota and fecal metabolites in mice. *Food Chem Toxicol.* (2021) 148:111946. 10.1016/j.fct.2020.111946 33359793

[54] Choi J Kim N Ju I Eo H Lim S Jang S Oral administration of *Proteus mirabilis* damages dopaminergic neurons and motor functions in mice. *Sci Rep.* (2018) 8:1275. 10.1038/s41598-018-19646-x 29352191 PMC5775305

[55] Cook R Fulcher J Tobin N Li F Lee D Woodward C Alterations to the gastrointestinal microbiome associated with methamphetamine use among young men who have sex with men. *Sci Rep.* (2019) 9:14840. 10.1038/s41598-019-51142-8 31619731 PMC6795845

[56] Lucerne K Osman A Meckel K Kiraly D. Contributions of neuroimmune and gut-brain signaling to vulnerability of developing substance use disorders. *Neuropharmacology.* (2021) 192:108598. 10.1016/j.neuropharm.2021.108598 33965398 PMC8220934

[57] Deng D Su H Song Y Chen T Sun Q Jiang H Altered fecal microbiota correlated with systemic inflammation in male subjects with methamphetamine use disorder. *Front Cell Infect Microbiol.* (2021) 11:783917. 10.3389/fcimb.2021.783917 34869080 PMC8637621

[58] MacNicol B. The biology of addiction. *Can J Anaesth.* (2017) 64:141–8. 10.1007/s12630-016-0771-2 27837404

[59] Dong Y Taylor J Wolf M Shaham Y. Circuit and synaptic plasticity mechanisms of drug relapse. *J Neurosci.* (2017) 37:10867–76. 10.1523/JNEUROSCI.1821-17.2017 29118216 PMC5678019

[60] Feltenstein M See R Fuchs R. Neural substrates and circuits of drug addiction. *Cold Spring Harb Perspect Med.* (2021) 11:a039628. 10.1101/cshperspect.a039628 32205414 PMC7923743

[61] Bardo M. Neuropharmacological mechanisms of drug reward: beyond dopamine in the nucleus accumbens. *Crit Rev Neurobiol.* (1998) 12:37–67. 10.1615/critrevneurobiol.v12.i1-2.30 9444481

[62] González-Arancibia C Urrutia-Piñones J Illanes-González J Martinez-Pinto J Sotomayor-Zárate R Julio-Pieper M Do your gut microbes affect your brain dopamine? *Psychopharmacology.* (2019) 236:1611–22. 10.1007/s00213-019-05265-5 31098656

[63] García-Cabrerizo R Carbia C Riordan K Schellekens H Cryan J. Microbiota-gut-brain axis as a regulator of reward processes. *J Neurochem.* (2021) 157:1495–524. 10.1111/jnc.15284 33368280

[64] Cornejo-Pareja I Martín-Núñez G Roca-Rodríguez M Cardona F Coin-Aragüez L Sánchez-Alcoholado L H. *pylori* eradication treatment alters gut microbiota and GLP-1 secretion in humans. *J Clin Med.* (2019) 8:451. 10.3390/jcm8040451 30987326 PMC6517938

[65] Alhadeff A Rupprecht L Hayes MR. GLP-1 neurons in the nucleus of the solitary tract project directly to the ventral tegmental area and nucleus accumbens to control for food intake. *Endocrinology.* (2012) 153:647–58. 10.1210/en.2011-1443 22128031 PMC3275387

[66] Tian L Jin T. The incretin hormone GLP-1 and mechanisms underlying its secretion. *J Diabetes.* (2016) 8:753–65. 10.1111/1753-0407.12439 27287542

[67] Breton J Tennoune N Lucas N Francois M Legrand R Jacquemot J Gut commensal E. coli proteins activate host satiety pathways following nutrient-induced bacterial growth. *Cell Metab.* (2016) 23:324–34. 10.1016/j.cmet.2015.10.017 26621107

[68] Egecioglu E Engel J Jerlhag E. The glucagon-like peptide 1 analogue, exendin-4, attenuates the rewarding properties of psychostimulant drugs in mice. *PLoS One.* (2013) 8:e69010. 10.1371/journal.pone.0069010 23874851 PMC3712951

[69] Harasta A Power J von Jonquieres G Karl T Drucker D Housley G Septal glucagon-like peptide 1 receptor expression determines suppression of cocaine-induced behavior. *Neuropsychopharmacology.* (2015) 40:1969–78. 10.1038/npp.2015.47 25669605 PMC4839521

[70] Schmidt H Mietlicki-Baase E Ige K Maurer J Reiner D Zimmer D Glucagon-like peptide-1 receptor activation in the ventral tegmental area decreases the reinforcing efficacy of cocaine. *Neuropsychopharmacology.* (2016) 41:1917–28. 10.1038/npp.2015.362 26675243 PMC4869061

[71] Havlickova T Charalambous C Lapka M Puskina N Jerabek P Sustkova-Fiserova M. Ghrelin receptor antagonism of methamphetamine-induced conditioned place preference and intravenous self-administration in rats. *Int J Mol Sci.* (2018) 19:2925. 10.3390/ijms19102925 30261633 PMC6213741

[72] Xue Y Luo Y Wu P Shi H Xue L Chen C A memory retrieval-extinction procedure to prevent drug craving and relapse. *Science.* (2012) 336:241–5. 10.1126/science.1215070 22499948 PMC3695463

[73] Luo Y Xue Y Liu J Shi H Jian M Han Y A novel UCS memory retrieval-extinction procedure to inhibit relapse to drug seeking. *Nat Commun.* (2015) 6:7675. 10.1038/ncomms8675 26169171 PMC4510700

[74] Santello M Toni N Volterra A. Astrocyte function from information processing to cognition and cognitive impairment. *Nat Neurosci.* (2019) 22:154–66. 10.1038/s41593-018-0325-8 30664773

[75] Araki T Ikegaya Y Koyama R. The effects of microglia- and astrocyte-derived factors on neurogenesis in health and disease. *Eur J Neurosci.* (2021) 54:5880–901. 10.1111/ejn.14969 32920880 PMC8451940

[76] Ma Q Xing C Long W Wang H Liu Q Wang R. Impact of microbiota on central nervous system and neurological diseases: the gut-brain axis. *J Neuroinflammation.* (2019) 16:53. 10.1186/s12974-019-1434-3 30823925 PMC6397457

[77] Wang Q Hu Y Wan J Dong B Sun J. Lactate: a novel signaling molecule in synaptic plasticity and drug addiction. *Bioessays.* (2019) 41:e1900008. 10.1002/bies.201900008 31270822

[78] Boury-Jamot B Halfon O Magistretti P Boutrel B. Lactate release from astrocytes to neurons contributes to cocaine memory formation. *Bioessays.* (2016) 38:1266–73. 10.1002/bies.201600118 27699812

[79] Zhang Y Xue Y Meng S Luo Y Liang J Li J Inhibition of lactate transport erases drug memory and prevents drug relapse. *Biol Psychiatry.* (2016) 79:928–39. 10.1016/j.biopsych.2015.07.007 26293178

[80] Boutrel B Magistretti PJ. A role for lactate in the consolidation of drug-related associative memories. *Biol Psychiatry.* (2016) 79:875–7. 10.1016/j.biopsych.2016.04.010 27198521

[81] Margineanu M Sherwin E Golubeva A Peterson V Hoban A Fiumelli H Gut microbiota modulates expression of genes involved in the astrocyte-neuron lactate shuttle in the hippocampus. *Eur Neuropsychopharmacol.* (2020) 41:152–9. 10.1016/j.euroneuro.2020.11.006 33191074

[82] Wang J He M Guo W Zhang Y Sui X Lin J Microbiome-metabolomics reveals endogenous alterations of energy metabolism by the dushen tang to attenuate D-Galactose-induced memory impairment in rats. *Biomed Res Int.* (2021) 2021:6649085. 10.1155/2021/6649085 34136571 PMC8175156

[83] Dietert R. Microbiome first medicine in health and safety. *Biomedicines.* (2021) 9:1099. 10.3390/biomedicines9091099 34572284 PMC8468398

[84] Dietert R. Microbiome first approaches to rescue public health and reduce human suffering. *Biomedicines.* (2021) 9:1581. 10.3390/biomedicines9111581 34829809 PMC8615664

[85] Spencer C McQuade J Gopalakrishnan V McCulloch J Vetizou M Cogdill A Dietary fiber and probiotics influence the gut microbiome and melanoma immunotherapy response. *Science.* (2021) 374:1632–40. 10.1126/science.aaz7015 34941392 PMC8970537

[86] Fu X Chen T Cai J Liu B Zeng Y Zhang X. The Microbiome-Gut-Brain Axis, a potential therapeutic target for substance-related disorders. *Front Microbiol.* (2021) 12:738401. 10.3389/fmicb.2021.738401 34690981 PMC8526971

